# Identification of Fungus Resistant Wild Accessions and Interspecific Hybrids of the Genus *Arachis*

**DOI:** 10.1371/journal.pone.0128811

**Published:** 2015-06-19

**Authors:** Marcos Doniseti Michelotto, Waldomiro Barioni, Marcos Deon Vilela de Resende, Ignácio José de Godoy, Eduardo Leonardecz, Alessandra Pereira Fávero

**Affiliations:** 1 Pólo Centro Norte, Agência Paulista de Tecnologia dos Agronegócios, Pindorama, São Paulo, Brazil; 2 Embrapa Southeast Livestock, São Carlos, São Paulo, Brazil; 3 Embrapa Forestry,Colombo, Paraná, Brazil; 4 Agronomic Institute of Campinas, Campinas, São Paulo, Brazil; 5 Laboratory of Scientific Computing, Campus Planaltina, University of Brasília, Planaltina, Federal District, Brasília; United States Department of Agriculture, UNITED STATES

## Abstract

Peanut, *Arachis hypogaea* L., is a protein-rich species consumed worldwide. A key improvement to peanut culture involves the development of cultivars that resist fungal diseases such as rust, leaf spot and scab. Over three years, we evaluated fungal resistance under field conditions of 43 wild accessions and three interspecific hybrids of the genus *Arachis*, as well as six *A*. *hypogaea* genotypes. In the first year, we evaluated resistance to early and late leaf spot, rust and scab. In the second and third years, we evaluated the 18 wild species with the best resistance scores and control cultivar IAC Caiapó for resistance to leaf spot and rust. All wild accessions displayed greater resistance than *A*. *hypogaea* but differed in their degree of resistance, even within the same species. We found accessions with as good as or better resistance than *A*. *cardenasii*, including: *A*. *stenosperma* (V15076 and Sv 3712), *A*. *kuhlmannii* (V 6413), *A*. *kempff-mercadoi* (V 13250), *A*. *hoehnei* (KG 30006), and *A*. *helodes* (V 6325). Amphidiploids and hybrids of *A*. *hypogaea* behaved similarly to wild species. An additional four accessions deserve further evaluation: *A*. *magna* (V 13751 and KG 30097) and *A*. *gregoryi* (V 14767 and V 14957). Although they did not display as strong resistance as the accessions cited above, they belong to the B genome type that is crucial to resistance gene introgression and pyramidization in *A*. *hypogaea*.

## Introduction

The oil and protein reach peanut **(** *Arachis hypogaea* L.) is consumed both *in natura* and processed as oil, constituting the fifth largest oleaginous crop worldwide [1]. his plant, which is native from South America, belongs to a genus with 81 described species distributed in nine taxonomic sections [2,3]. The *Arachis* section includes 31 species including the commercial peanut.

The development of fungus resistance represents one of the main challenges for the improvement of cultivated peanuts. Some of the most severe fungal foliar diseases include leaf spot (*Cercosporidium personatum* Berk & Curtis Deighton and *Cercospora arachidicola* Horii), rust (*Puccinia arachidis* Speg.), web blotch (*Phoma arachidicola* Marasas, Pauer & Boerema), and scab (*Sphaceloma arachidis* Bit & Jenk). The genus *Arachis* has long been studied with regards to the introgression potential of resistance genes in peanut cultivars [4–9]. Extensive studies have shown that the *A*. *cardenasii* accession GKP 10017 is resistant to diseases [10,8]. However, these studies were conducted in greenhouses or laboratories, with detached leaves. Obstacles associated with field research such as the low availability of seeds of wild species, analytical difficulties, and inoculum natural pressure are common in wild *Arachis* bioassays. Field studies of ancient and recently-collected accessions are necessary, especially in areas close to production centers.

The state of São Paulo accounts for 80% of peanut production in Brazil. Leading phytosanitary threats in the state include late leaf spot (*Cercosporidium personatum*), early leaf spot (*Cercospora arachidicola*), rust (*Puccinia arachidis*), and scab (*Sphaceloma arachidis*). Furthermore, inoculum pressure in São Paulo is consistently high [9], making this a good site for the assessment of genotype resistance to prevailing pathogens.

We evaluated 43 accessions and three interspecific *Arachis* hybrids with regards to resistance to foliar diseases under field conditions in the state of São Paulo. Accessions might be later crossed generating amphidiploids (artificially doubled interspecific hybrids with distinct genomic backgrounds that might be AABB or might have other genomic combinations) to be further crossed with cultivars or elite lines of *A*. *hypogaea*, generating segregated populations that can be selected and backcrossed in a breeding program.

## Materials and Methods

### Plant culture

Bioassays were conducted at the Pólo Apta Centro Norte experimental area in Pindorama, São Paulo, Brazil. Seeds were originally provided by the *Arachis* Germplasm Bank, Embrapa Genetic Resources and Biotechnology. Seeds of different genotypes (Table 1) were treated with the fungicide Plantacol^®^ (10g/100kg of seeds) and germinated in paper towels in a room with adequate temperature, air humidity and light. Seedlings were transplanted to 200-ml plastic cups filled with soil and sand (3:1) and placed in a greenhouse. When plants reached a height of 10 to 15 cm they were transplanted to the field in soil previously prepared with 250 kg/ha of 8-28-16 NPK.

**Table 1:** *Arachis* spp. accessions included in the present study.

Accessions Code	Species	Brazilian Accessions Code	Collection sitesCity	State in Brazil or Country	Lat(W)	Long(S)	Alt(m)	Genome
K 9484	*A*. *batizocoi* Krapov. & W. C. Gregory	013315	Parapeti	BOL	20° 05’	63° 14’	700	K
KG 35005	*A*. *benensis* Krapov. & W.C. Gregory	037206	Trinidad	BOL				F
GKP 10017	*A*. *cardenasii* Krapov. & W. C. Gregory	013404	Roboré	BOL	18° 20’	59° 46’	200	A
K 7988	*A*. *duranensis* Krapov. & W. C. Gregory	013307	Campo Duran	ARG	22° 19’	63° 13’	500	A
VSGr 6389	*A*. *gregoryi* C. E. Simpson, Krapov.& Valls	012696	Vila Bela da Ssa. Trindade	MT	15° 19’	60° 06’	210	B
VOfSv 14760	*A*. *gregoryi* C. E. Simpson, Krapov.& Valls	038792	Vila Bela da Ssa. Trindade	MT	16° 08’	59° 47’		B
VOfSv 14767	*A*. *gregoryi* C. E. Simpson, Krapov.& Valls	038814	Vila Bela da Ssa. Trindade	MT	16° 05’	59° 58’	290	B
VS 14957	*A*. *gregoryi* C. E. Simpson, Krapov.& Valls	040002	Vila Bela da Ssa. Trindade	MT	15° 22’	60° 14’		B
CoSzSv 6862	*A*. *helodes* Martius ex Krapov & Rigoni	018619		MT	15° 22’	56° 13’	175	A
VSGr 6325	*A*. *helodes* Martius ex Krapov & Rigoni	012505	S. Antonio do Leverger	MT	15° 52’	56° 04’	150	A
KG 30006	*A*. *hoehnei* Krapov. & W. C. Gregory	036226	Corumbá	MS	18° 15’	57° 28’		A
VRcMmSv 14546	*A*. *hoehnei* Krapov. & W. C. Gregory	022641	Corumbá	MS	19 ° 15’	57 ° 22’	100	A
cv. BR1	*A*. *hypogaea subsp*. *fastigiata var*. *fastigiata*	033383						AB
cv. IAC Caiapó	*A*. *hypogaea*	037371						AB
2562	*A*. *hypogaea*	037354						AB
IAC Runner 886	*A hypogaea subsp*. *hypogaea var*. *hypogaea*	037389						AB
cv. IAC Tatu-ST	*A*. *hypogaea subsp*. *fastigiata var*. *fastigiata*	011606	Campinas	SP				AB
V 12549	*A*. *hypogaea subsp*. *hypopaea var*. *hypogaea*	030716						AB
KGPScS 30076	*A*. *ipaënsis* Krapov. & W. C. Gregory	036234	Ipa	BOL	21° 00’	63° 25’	650	B
V 13250	*A*. *kempff-mercadoi* Krapov., W. C. Gregory & C. E. Simpson	030643	Sta. Cruz de la Sierra	BOL	17° 45’	63° 10’	280	A
VKSSv 8979	*A*. *kuhlmannii* Krapov. & W. C. Gregory	020354	Cáceres	MT	15° 35’	57° 13’	210	A
VPoBi 9243	*A*. *kuhlmannii* Krapov. & W. C. Gregory	022560	Corumbá	MS	18° 52’	56° 16’	100	A
VPoJSv 10506	*A*. *kuhlmannii* Krapov. & W. C. Gregory	024953	N. Sra. do Livramento	MT	15° 48’	56° 21’		A
VRGeSv 7639	*A*. *kuhlmannii* Krapov. & W. C. Gregory	017515	Miranda	MS	20° 15’	56° 23’	125	A
VSGr 6351	*A*. *kuhlmannii* Krapov. & W. C. Gregory	012602	Cáceres	MT	15° 56’	57° 48’	130	A
VSGr 6413	*A*. *kuhlmannii* Krapov. & W. C. Gregory	012688	Cáceres	MT	15° 47’	57° 25’	200	A
VSW 9912	*A*. *kuhlmannii* Krapov. & W. C. Gregory	022900	Aquidauana	MS	20° 26’	55° 54’	210	A
KGSSc 30097	*A*. *magna* Krapov., W. C. Gregory & C. E. Simpson	036871	San Ignacio de Velasco	BOL	16° 22’	60° 58’	370	B
VPzSgRcSv 13761	*A*. *magna* Krapov., W. C. Gregory & C. E. Simpson	036218	Vila Bela da Ssa. Trindade	MT	15° 21’	60° 04’	380	B
VSPmSv 13751	*A*. *magna* Krapov., W. C. Gregory & C. E. Simpson	033812	Vila Bela da Ssa Trindade	MT	16° 16’	59° 27’	530	B
VOa 14165	*A*. *monticola* Krapov. & Rigoni	036188	Yala, Jujuy	ARG	24° 07’	65° 23’		AB
VSPmSv 13710	*A*. *simpsonii* Krapov. & W. C. Gregory	033685	Porto Esperidião	MT	15° 58’	58° 31’	270	A
HLK 408	*A*. *stenosperma* Krapov. & W. C. Gregory	013366	Antonina	PR	25° 24’	48° 44’	3	A
Lm 5	*A*. *stenosperma* Krapov. & W. C. Gregory	036013	Antonina	PR				A
SvW 3712	*A*. *stenosperma* Krapov. & W. C. Gregory	035254	Cocalinho	MT	14° 22’	51° 00’	220	A
VSStGdW 7805-AR	*A*. *stenosperma* Krapov. & W. C. Gregory	032476	São Felix do Araguaia	MT	11° 38’	50° 48’	240	A
VKSSv 9010	*A*. *stenosperma* Krapov. & W. C. Gregory	020176	Santo Antonio do Leverger	MT	15° 52’	56° 04’	150	A
VMiSv 10229	*A*. *stenosperma* Krapov. & W. C. Gregory	023001	Cananéia	SP	25° 01’	47° 55’	10	A
VS 13670	*A*. *stenosperma* Krapov. & W. C. Gregory	018104	Araguaiana	MT	15° 33’	52° 12’	350	A
VSMGeSv 7379	*A*. *stenosperma* Krapov. & W. C. Gregory	016063	Antonina	PR	25° 26’	48° 42’	3	A
VSPmSv 13832	*A*. *stenosperma* Krapov. & W. C. Gregory	033961	S. M. do Araguaia/Luiz Alves	MT	13° 13’	50° 34’	280	A
VSPmW 13824	*A*. *stenosperma* Krapov. & W. C. Gregory	033936	S. M. do Araguaia/Luiz Alves	MT	13° 13’	50° 34’	280	A
VSSv 13258	*A*. *stenosperma* Krapov. & W. C. Gregory	016128	São Sebastião	SP	23° 45’	45° 24’	5	A
VSv 10309	*A*. *stenosperma* Krapov. & W. C. Gregory	024830	Rondonópolis	MT	16° 28’	54° 39’	215	A
VArLf 15076	*A*. *stenosperma* Krapov. & W. C. Gregory	040266	Matinhos	PR				A
WPz 421	*A*. *stenosperma* Krapov. & W. C. Gregory	033511	Alvorada	TO	12° 36’	49° 20’	310	A
WiDc 1118	*A*. *williamsii* Krapov. & W. C. Gregory	036897	Trinidad	BOL				B
An 2 = (V 6389 x V 9401)^4x^	(*A*. *gregoryi* x *A*. *linearifolia*)^4x^							AB
An 4 = (KG 30076 x V 14167)^4x^	(*A*.*ipaënsis* x *A*. *duranensis*)^4x^							AB
IAC Caiapó x An2	*A*. *hypogaea* x (*A*. *gregoryi* x *A*. *linearifolia*)^4x^							AB

* Collectors: = Ar = A.R. Custodio, Bi = L. B. Bianchetti, Co = L. Coradin, Dc = D. Claure, G = W. C. Gregory, Gd = I. J. Godoy, Ge = M. A. N. Gerin, Gr = A. Gripp, H = R. Hammons, J = L. Jank, K = A. Krapovickas, L = W.R. Langford, Lf = L. G. Faria,Lm = L. Monçato, M = J. P. Moss, Mi = S.T.S.Miotto, Mm = M. Moraes, Oa = O.Ahumada, Of = F. O. Freitas, P = J. R. Pietralli, Pm = R. N. Pittmann, Po = A. Pott, Pz = E. Pizarro, R = V. R. Rao, Rc = R.C.Oliveira, S = C. E. Simpson, Sc = A. Schinini, Sg = A. K. Singh, St = H. T. Stalker, Sv = G. P. Silva, Sz = R. Schultze-Kraft, V = J. F. M. Valls, W = W. L. Werneck, Wi = D. E. Williams.

During the first year, we evaluated 43 accessions belonging to 10 wild species, six *A*. *hypogaea* genotypes and three interspecific hybrids, including amphidiploids and segregating populations (Table 1). Twenty-five F_2_ individuals of the progenie by the cross between IAC Caiapó and the amphidiploid An 2 were evaluated. The average of the experimental unit were used for analyses of variance. In the second and third years, we selected the 18 most resistant accessions and the IAC Caiapó cultivar as control.

The experiment design was random uncompleted delineated block with four replications. Each block was initially composed of four meters with five plants spaced one meter apart and with a separation of 1.5 meters between lines. This spacing was needed because of the ample growth of these plant species. Just three plants in the middle of the experimental unit were evaluated. Every block was sprayed twice-monthly with insecticides to avoid infestation. Weed control was performed with the pre-transplantation application of commercially available Trifluralin (2.5 l/ha). During plant growth, weed control was performed manually. The experimental design was the same for all three years of evaluations apart from the number of accessions analyzed.

### Resistance testing

In the first year, fungal diseases evaluated included early leaf spot (*Cercospora arachidicola*), late leaf spot (*Cercosporidium personatum*), rust (*Puccinia arachidis*.) and scab (*Sphaceloma arachidis*). All diseases except for scab were also evaluated in the second and third years to confirm resistance of wild species accessions (18 genotypes and IAC Caiapó control). Scab was not evaluated in the second and third years due to its low incidence. A 1–9 visual grade scale for damage caused at the end of the plant cycle was used in all evaluations.

Resistance data for early leaf spot, late leaf spot and rust were analyzed following the SAS GLM procedure [11] taking into account the model cultivar effect (1 to 19) and time (years 1, 2 and 3). Data from early and late leaf spot were transformed 1/x and log10(x), respectively, as suggested for the normalization of residues and cultivar variance homogeneity. In the comparison of averages from cultivars, we adopted Duncan’s test at a significance of 5%. Software Selegen-Reml/Blup [12] were used for Restricted Maximum Likelihood/ Best, Linear, Unbiased Prediction (REML/BLUP) analysis (Model 20 for first year data and Model 29 for three-year data).

Data were also subjected to grouping analysis (GA) complemented with principal component analysis (PCA) to group genotypes according to the variables: late leaf spot, early leaf spot, scab, and rust. Genotype GA was performed according to Ward’s method [13], and Euclidian distance was considered a measure of dissimilarity. Dendrogram and connection graphs were used to interpret GA results. In PCA the two first principal components (PC1 and PC2) were considered the most important in their respective contributions to total variability. PC1 and PC2 allowed for simultaneous visualization of variable and genotype projections as well as deduction of the linear correlation among the variables: late leaf spot, early leaf spot, scab and rust. The software used for PCA and GA was STATISTICA [14], other analyses were conducted with SAS [11] and MS Office Excel.

Analysis of Variance conducted on data for the three different years showed significant differences between years for all three diseases and the interaction accessions x years for disease (late leaf spot and rust). Therefore, average measurements were used for GA.

## Results and Discussion

In the first year of study, we evaluated resistance to late leaf spot, early leaf spot, rust and scab. Within 50 accessions evaluated at the first year (Table 2) there was a large difference in resistance to late leaf spot, with averages ranging from 1.75 to 9. On the other side, for early leaf spot, scab and rust, the variation among wild accessions was less significant. It is possible to verify either the difficulty to select accessions based on ANOVA and Duncan test. These results justify the utilization of PCA and grouping analysis.

**Table 2:** Duncan test results for *Arachis* spp. accessions for resistance to late leaf spot (LLS), early leaf spot (ELS), scab (S) and rust (R) in field assay (first year).

Accessions Code	Species	LLS	ELS	S	R
2562	*A*. *hypogaea*	9.00 a^1^	-	-	5.50 b
IAC Tatu-ST	*A*. *hypogaea*	8.75 a	2.00 efg	3.00 a	5.00 bc
IAC Runner 886	*A hypogaea*	8.69 a	1.67 efgh	2.33 abc	8.00 a
BR1	*A*. *hypogaea*	8.00 ab	5.00 a	2.50 ab	4.67 cd
IAC Caiapó	*A*. *hypogaea*	7.50 bc	5.19 a	2.44 ab	4.17 d
K 35005	*A*. *benensis*	6.75 c	1.00 h	1.00 e	1.00 f
K 9484	*A*. *batizocoi*	5.33 d	1.67 efgh	1.67 bcde	1.00 f
K 7988	*A*. *duranensis*	5.33 d	3.67 b	1.00 e	1.00 f
V 14165	*A*. *monticola*	5.33 d	1.33 fgh	2.33 abc	2.00 e
V 12549	*A*. *hypogaea*	5.00 de	3.25 bc	2.50 ab	5.00 bc
V 13761	*A*. *magna*	5.00 de	1.00 h	1.00 e	1.33 f
K 30097	*A*. *magna*	4.67 def	1.00 h	1.33 de	1.33 f
V 7805-AR	*A*. *stenosperma*	4.67 def	1.33 fgh	1.00 e	1.00 f
An 4	(*A*.*ipaënsis* x *A*. *duranensis*)^4x^	4.50 defg	1.75 efgh	1.50 cde	1.00 f
V 10506	*A*. *kuhlmannii*	4.00 efgh	2.25 def	1.00 e	1.33 f
V 8979	*A*. *kuhlmannii*	4.00 efgh	3.00 bcd	1.00 e	1.50 ef
V 14767	*A*. *gregoryi*	4.00 efgh	1.50 fgh	1.25 de	1.00 f
V 7639	*A*. *kuhlmannii*	4.00 efgh	1.33 fgh	1.00 e	1.33 f
IAC Caiapó x An2	*A*. *hypogaea* x (*A*. *gregoryi* x *A*. *linearifolia*)^4x^	3.78 efghi	1.63 efgh	1.48 de	1.55 ef
K 30076	*A*. *ipaënsis*	3.75 efghi	1.25 gh	2.50 ab	2.00 e
V 9243	*A*. *kuhlmannii*	3.75 efghi	1.25 gh	1.00 e	1.00 f
V 6351	*A*. *kuhlmannii*	3.75 efghi	2.25 def	1.25 de	1.00 f
An 2	(*A*. *gregoryi* x *A*. *linearifolia*)^4x^	3.75 efghi	1.00 h	2.00 bcd	1.00 f
W 421	*A*. *stenosperma*	3.50 fghij	2.00 efg	1.00 e	1.00 f
Wi 1118	*A*. *williamsii*	3.50 fghij	1.50 fgh	1.00 e	2.00 e
V 14546	*A*. *hoehnei*	3.50 fghij	2.50 cde	1.25 ed	1.00 f
V 13832	*A*. *stenosperma*	3.33 ghijk	1.33 fgh	1.33 de	1.00 f
Co 6862	*A*. *helodes*	3.25 ghijk	1.75 efgh	1.00 e	1.00 f
V 9912	*A*. *kuhlmannii*	3.25 ghijk	1.00 h	1.00 e	1.00 f
V 13751	*A*. *magna*	3.25 ghijk	1.25 gh	1.00 e	1.00 f
V 14957	*A*. *gregoryi*	3.00 hijkl	1.00 h	1.25 de	1.00 f
V 6389	*A*. *gregoryi*	3.00 hijkl	1.00 h	1.75 bcde	1.00 f
H 408	*A*. *stenosperma*	3.00 hijkl	1.67 efgh	1.33 de	1.00 f
V 13824	*A*. *stenosperma*	3.00 hijkl	1.67 efgh	1.33 de	1.00 f
V 10309	*A*. *stenosperma*	3.00 hijkl	1.67 efgh	1.67 bcde	1.50 ef
V 14760	*A*. *gregoryi*	2.75 hijkl	1.00 h	1.50 cde	1.00 f
G 10017	*A*. *cardenasii*	2.67 hijkl	1.67 efgh	1.00 e	1.33 f
V 13710	*A*. *simpsonii*	2.67 hijkl	2.00 efg	1.00 e	1.00 f
V 7379	*A*. *stenosperma*	2.67 hijkl	1.67 efgh	1.67 bcde	1.33 f
V 13670	*A*. *stenosperma*	2.50 ijkl	2.00 efg	1.50 cde	1.00 f
V 15076	*A*. *stenosperma*	2.33 jkl	1.67 efgh	1.00 e	1.00 f
V 6325	*A*. *helodes*	2.33 ijkl	1.33 fgh	1.00 e	1.33 f
Lm 5	*A*. *stenosperma*	2.33 jkl	1.67 efgh	1.33 de	1.00 f
V 9010	*A*. *stenosperma*	2.33 jkl	1.67 efgh	1.33 de	1.00 f
V 10229	*A*. *stenosperma*	2.25 jkl	1.75 efgh	1.50 cde	1.00 f
V 13258	*A*. *stenosperma*	2.25 jkl	1.50 fgh	1.50 cde	1.00 f
V 13250	*A*. *kempff-mercadoi*	2.00 kl	1.50 fgh	1.00 e	1.00 f
V 6413	*A*. *kuhlmannii*	2.00 kl	1.67 efgh	1.00 e	1.00 f
Sv 3712	*A*. *stenosperma*	2.00 kl	1.00 h	1.00 e	1.00 f
K 30006	*A*. *hoehnei*	1.75 l	1.25 gh	1.25 ed	1.00 f

^1.^Distinct letters indicate significant differences among accessions according to Duncan’s test (p<0,05)

REML/BLUP analysis were shown in Table 3 for 50 genotypes in first year field assay. The selection accuracy of genotypes had a high value, as well as PEV value was low for all variables. All CV_gi%_ were higher than CV_e%_ values except for scab variable indicating that the environment had a important effect in the phenotypic pattern of this disease.

**Table 3:** Estimatives of components of variance (Individual REML) and the components of average (Individual BLUP) for the variables resistance to late leaf spot (LLS), early leaf spot (ELS), scab (S) and rust (R) and 50 genotypes, in first year of field assay.

	Components of average (Individual BLUP)																				
Genotype	LLS					ELS					S					R					
	Rank	g	u + g	GG	Na	Rank	g	u + g	GG	Na	Rank	g	u + g	GG	Na	Rank	g	u + g	GG	Na	GR
2562	1	4.8916	8.8054	4.8916	8.8054	-	-	-	-	-	-	-	-	-	-	2	3.7621	5.4159	4.9741	6.6279	3
IAC Tatu-ST	2	4.6512	8.565	4.7714	8.6852	6	1.0018	2.7828	1.7215	3.5024	1	1.0319	2.4708	1.0319	2.4708	4	3.1440	4.7979	4.0967	5.7505	13
IAC Runner 866	3	4.5916	8.5054	4.7115	8.6253	5	1.0018	2.7828	1.8654	3.6464	7	0.645	2.0839	0.7881	2.227	1	6.1861	7.8400	6.1861	7.8400	16
BR 1	4	3.8618	7.7756	4.4991	8.4129	2	2.5442	4.3252	2.7551	4.5360	5	0.7247	2.1636	0.8361	2.2749	8	0.3137	1.9676	2.8104	4.4643	19
IAC Caiap00F3	5	3.4490	7.3628	4.2890	8.2029	1	2.9659	4.7469	2.9659	4.7469	4	0.7758	2.2147	0.8639	2.3028	6	2.4749	4.1287	3.6381	5.2920	16
V 12549	10	1.0446	4.9585	2.9179	6.8318	4	1.2788	3.0598	2.0813	3.8623	2	0.8239	2.2628	0.9279	2.3668	3	3.2945	4.9484	4.4142	6.0681	19
K 9484	7	1.3206	5.2344	3.6419	7.5557	19	-0.1502	1.6308	0.6575	2.4384	10	0.2026	1.6415	0.6397	2.0786	20	-0.6438	1.0101	0.9696	2.6234	56
V 14165	9	1.3206	5.2344	3.1261	7.0399	35	-0.4327	1.3483	0.2428	2.0238	6	0.6916	2.1305	0.8120	2.2509	7	0.3408	1.9947	3.1671	4.8209	57
V 8979	18	-0.0382	3.8756	1.7945	5.7083	7	0.8747	2.6557	1.6005	3.3815	31	-0.2428	1.1961	0.1809	1.6198	11	-0.1930	1.4608	2.0403	3.6941	67
IAC Caiapó x An 2	19	-0.1325	3.7813	1.693	5.6068	18	-0.1349	1.6461	0.7024	2.4833	18	0.0288	1.4677	0.3939	1.8328	10	-0.1441	1.5098	2.2636	3.9175	65
K 30076	20	-0.1575	3.7563	1.6005	5.5143	38	-0.4622	1.3187	0.1880	1.9689	3	0.8239	2.2628	0.8933	2.3321	5	2.9672	4.6210	3.8708	5.5246	66
K 7988	8	1.3206	5.2344	3.3518	7.2656	3	1.5363	3.3172	2.3488	4.1297	34	-0.2883	1.1506	0.1395	1.5784	22	-0.6438	1.0101	0.8229	2.4768	67
V 10309	35	-0.976	2.9378	0.6628	4.5766	17	-0.1218	1.6592	0.7516	2.5326	12	0.1776	1.6164	0.5647	2.0036	12	-0.1930	1,4608	1,8542	3,508	76
An 4	14	0.5638	4.4776	2.2944	6.2082	15	-0.0269	1.7540	0.8617	2.6427	15	0.0475	1.4863	0.4645	1.9033	34	-0.6438	1.0101	0.3052	1.9591	78
V 14546	24	-0.3980	3.5158	1.2975	5.2113	8	0.6260	2.4069	1.4787	3.2596	27	-0.1467	1.2922	0.2366	1.6755	26	-0.6438	1.0101	0.5973	2.2511	85
V 10506	16	0.0829	3.9967	2.0179	5.9318	9	0.4083	2.1893	1.3598	3.1407	47	-0.3408	1.0981	0.0145	1.4534	16	-0.3164	1.3375	1.3115	2.9654	88
Wi 1118	26	-0.5071	3.4067	1.1629	5.0767	31	-0.3273	1.4536	0.3300	2.1109	30	-0.2428	1.1961	0.1951	1.6339	9	0.2967	1.9506	2.5311	4.185	96
V 7379	39	-1.2182	2.6956	0.4724	4.3862	27	-0.1502	1.6308	0.4182	2.1991	11	0.2026	1.6415	0.5999	2.0388	19	-0.3164	1,3375	1,0545	2,7083	96
V 7639	17	0.0512	3.9650	1.9023	5.8161	32	-0.4327	1.3483	0.3062	2.0871	33	-0.2883	1.1506	0.1525	1.5914	14	-0.3164	1.3375	1.5441	3.1979	96
V 6351	22	-0.1575	3.7563	1.4407	5.3545	10	0.4083	2.1893	1.2646	3.0456	29	-0.1467	1.2922	0.2102	1.6490	36	-0.6438	1.0101	0.2525	1.9064	97
K 30097	13	0.6871	4.6009	2.4275	6.3413	48	-0.713	1.0679	0.0149	1.7958	20	-0.0438	1.3951	0.3502	1.789	18	-0.3164	1.3375	1.1306	2.7845	99
V 14767	15	0.0829	3.9967	2.1469	6.0608	28	-0.2446	1.5364	0.3945	2.1755	28	-0.1467	1.2922	0.2229	1.6618	29	-0.6438	1.0101	0.4689	2.1227	100
GKP 10017	37	-1.2182	2.6956	0.5638	4.4776	20	-0.1502	1.6308	0.6171	2.3980	32	-0.2883	1.1506	0.1663	1.6052	13	-0.3164	1,3375	1,6872	3,3411	102
V 7805-AR	12	0.6871	4.6009	2.5725	6.4863	33	-0.4327	1.3483	0.2838	2.0647	35	-0.2883	1.1506	0.1273	1.5662	27	-0.6438	1.0101	0.5513	2.2051	107
V 13832	27	-0.5847	3.3291	1.0981	5.0119	36	-0.4327	1.3483	0.2241	2.0050	24	-0.0438	1.3951	0.2845	1.7234	48	-0.6438	1.0101	0.0285	1.6823	135
K 35005	6	2.7277	6.6415	4.0288	7.9426	41	-0.6798	1.1011	0.1351	1.9160	42	-0.3408	1.0981	0.0568	1.4957	21	-0.6438	1.0101	0.8927	2.5466	110
Co 6862	28	-0.6384	3.2754	1.0361	4.9499	14	-0.0269	1.7540	0.9252	2.7062	43	-0.3408	1.0981	0.0476	1.4864	28	-0.6438	1,0101	0,5086	2,1625	113
V 13761	11	1.0039	4.9177	2.7439	6.6577	47	-0.713	1.0679	0.0303	1.8113	39	-0.2883	1.1506	0.0847	1.5236	17	-0.3164	1.3375	1.2158	2.8696	114
V 13670	40	-1.445	2.4689	0.4245	4.3383	13	0.0734	1.8543	0.9985	2.7794	13	0.0951	1.5340	0.5286	1.9675	49	-0.6828	0,9710	0,0139	1,6678	115
V 6389	32	-0.8789	3.035	0.8118	4.7256	44	-0.6798	1.1011	0.0795	1.8605	9	0.2416	1.6805	0.6882	2.1271	30	-0.6438	1,0101	0,4318	2,0856	115
V 14760	36	-1.1193	2.7945	0.6133	4.5271	43	-0.6798	1.1011	0.0972	1.8781	14	0.0475	1.4863	0.4942	1.9331	24	-0.6438	1,0101	0,7007	2,3545	117
HLK 408	33	-0.9014	3.0124	0.7599	4.6737	24	-0.1502	1.6308	0.4892	2.2702	21	-0.0438	1.3951	0.3314	1.7703	41	-0.6438	1,0101	0,1432	1,7971	119
V 10229	45	-1.6002	2.3136	0.2051	4.1189	16	-0.0269	1.754	0.8062	2.5871	16	0.0475	1.4863	0.4384	1.8773	42	-0.6438	1,0101	0,1245	1,7783	119
V 14957	31	-0.8789	3.035	0.8663	4.7801	42	-0.6798	1.1011	0.1157	1.8966	25	-0.1467	1.2922	0.2673	1.7061	23	-0.6438	1,0101	0,7591	2,4130	121
Lm 5	43	-1.5373	2.3765	0.2876	4.2014	23	-0.1502	1.6308	0.5170	2.2980	19	-0.0438	1.3951	0.3709	1.8098	39	-0.6438	1,0101	0,1836	1,8374	124
W 421	25	-0.3980	3.5158	1.2297	5.1435	11	0.1302	1.9111	1.1615	2.9424	49	-0.3408	1.0981	0.0000	1.4389	40	-0.6438	1.0101	0.1629	1.8168	125
V 6325	41	-1.5373	2.3765	0.3766	4.2904	34	-0.4327	1.3483	0.2627	2.0436	36	-0.2883	1.1506	0.1158	1.5547	15	-0.3164	1,3375	1,4200	3,0739	126
An 2	23	-0.1575	3.7563	1.3712	5.2850	46	-0.6798	1.1011	0.0465	1.8274	8	0.4357	1.8746	0.7441	2.1830	50	-0.6828	0.9710	0.0000	1.6539	127
V 13824	34	-0.9014	3.0124	0.711	4.6248	26	-0.1502	1.6308	0.4400	2.2210	23	-0.0438	1.3951	0.2988	1.7377	46	-0.6438	1,0101	0,0577	1,7115	129
V 9010	44	-1.5373	2.3765	0.2461	4.1599	25	-0.1502	1.6308	0.4637	2.2446	22	-0.0438	1.3951	0.3143	1.7532	43	-0.6438	1,0101	0,1066	1,7605	134
V 13710	38	-1.2182	2.6956	0.5169	4.4307	12	0.1302	1.9111	1.0756	2.8565	40	-0.2883	1.1506	0.0754	1.5142	44	-0.6438	1,0101	0,0896	1,7434	134
V 9243	21	-0.1575	3.7563	1.5168	5.4306	39	-0.4622	1.3187	0.1713	1.9522	45	-0.3408	1.0981	0.0303	1.4692	32	-0.6438	1.0101	0.3646	2.0184	137
V 13258	46	-1.6002	2.3136	0.1659	4.0797	30	-0.2446	1.5364	0.3519	2.1328	17	0.0475	1.4863	0.4154	1.8543	45	-0.6438	1,0101	0,0733	1,7271	138
K 30006	50	-2.081	1.8328	0.0000	3.9138	37	-0.4622	1.3187	0.2055	1.9865	26	-0.1467	1.2922	0.2513	1.6902	25	-0.6438	1,0101	0,6469	2,3008	138
V 15076	42	-1.5373	2.3765	0.3311	4.2449	22	-0.1502	1.6308	0.5474	2.3283	38	-0.2883	1.1506	0.0945	1.5334	38	-0.6438	1,0101	0,2054	1,8592	140
V 6413	48	-1.854	2.0598	0.082	3.9958	21	-0.1502	1.6308	0.5806	2.3615	37	-0.2883	1.1506	0.1048	1.5437	35	-0.6438	1,0101	0,2781	1,9320	141
V 9912	29	-0.6384	3.2754	0.9784	4.8922	45	-0.6798	1.1011	0.0626	1.8436	46	-0.3408	1.0981	0.0222	1.4611	33	-0.6438	1,0101	0,3340	1,9879	153
V 13751	30	-0.6384	3.2754	0.9245	4.8383	40	-0.4622	1.3187	0.1555	1.9364	48	-0.3408	1.0981	0.0071	1.446	37	-0.6438	1,0101	0,2283	1,8822	155
V 13250	47	-1.8406	2.0732	0.1232	4.037	29	-0.2446	1.5364	0.3725	2.1534	44	-0.3408	1.0981	0.0387	1.4776	31	-0.6438	1,0101	0,3971	2,0509	151
Sv 3712	49	-1.854	2.0598	0.0425	3.9563	49	-0.7130	1.0679	0.0000	1.7809	41	-0.2883	1.1506	0.0665	1.5054	47	-0.6438	1,0101	0,0428	1,6966	186
Components of variance (Individual REML)	Vg = 3.2625					Vg = 0.7196					Vg = 0.1900					Vg = 2.1206					
	Ve = 0.6488					Ve = 0.5351					Ve = 0.2735					Ve = 0.1328					
	Vf = 3.9113					Vf = 1.2547					Vf = 0.4635					Vf = 2.2535					
	h^2^_g_ = 0.8341 +- 0.1734					h^2^_g_ = 0.5735 +- 0.1464					h^2^_g_ = 0.4100 +- 0.1238					h^2^_g_ = 0.9411 +- 0.2006					
	h2mc = 0.9618					h2mc = 0.8705					h2mc = 0.7765					h2mc = 0.9846					
	Acclon = 0.9807					Acclon = 0.9330					Acclon = 0.8812					Acclon = 0.9923					
	CVgi% = 46.1507					CVgi% = 47.6306					CVgi% = 30.2939					CVgi% = 88.0511					
	CVe% = 20.5798					CVe% = 41.0749					CVe% = 36.3435					CVe% = 22.0374					
	CVr = 2.2425					CVr = 1.1596					CVr = 0.83355					CVr = 3.9955					
	PEV = 0.1248					PEV = 0.09317					PEV = 0.04247					PEV = 0.0327					
	SEP = 0.3533					SEP = 0.3052					SEP = 0.20608					SEP = 0.1808					
	GA = 3.9138					GA = 1.7809					GA = 1.4389					GA = 1.6539					

* g: genotypic efect, u + g: genotypic average, GG: genetic gain, Na = new average, GR: general rank, Vg: genotypic variance, Ve: residual variance, Vf: phenotypic variance, h^2^_g_ = plot heritability in broad sense, h2mc = average genotype heritability, Acclon = sellection genotype accuracy, CVgi% = individual coefficient of additive variance, CVe% = coefficient of experimental variation, CVr = coefficient of relative variation, PEV = prediction error variance of genotypic values, SEP = standard deviation of genotypic value, GA = general average

Resistance rank of each accession was obtained in individual BLUP analysis, as well as a general rank was observed by the sum of all ranks of the three diseases. The highest values are those with best resistance to the three diseases. Ranks of genotypes in BLUP analysis were very similar to Duncan test results (Table 2).

Genetic correlation between the variables LLS, ELS, S and R for the first year assay and for three years data, based on the REML/BLUP analysis, are shown in Table 4. In the first year, LLS and R was genetically correlated. In the analysis of three years for the 18 wild accessions selected as resistant and the control, all variables were correlated.

**Table 4:** Genetic correlation between variables (resistances to late and early leaf spots–LLS, ELS–scab–S, and rust—R) in first year for 50 accessions and in three years field assay for 18 selected accessions and one control.

	Genetic correlation				
	1st year			3 years	
Variable	LLS	ELS	S	LLS	ELS
LLS					
ELS	0.3986			0.9519	
S	0.2779	0.5447			
R	0.7058	0.3095	0.4062	0.9615	0.9801

**Fig 1:**
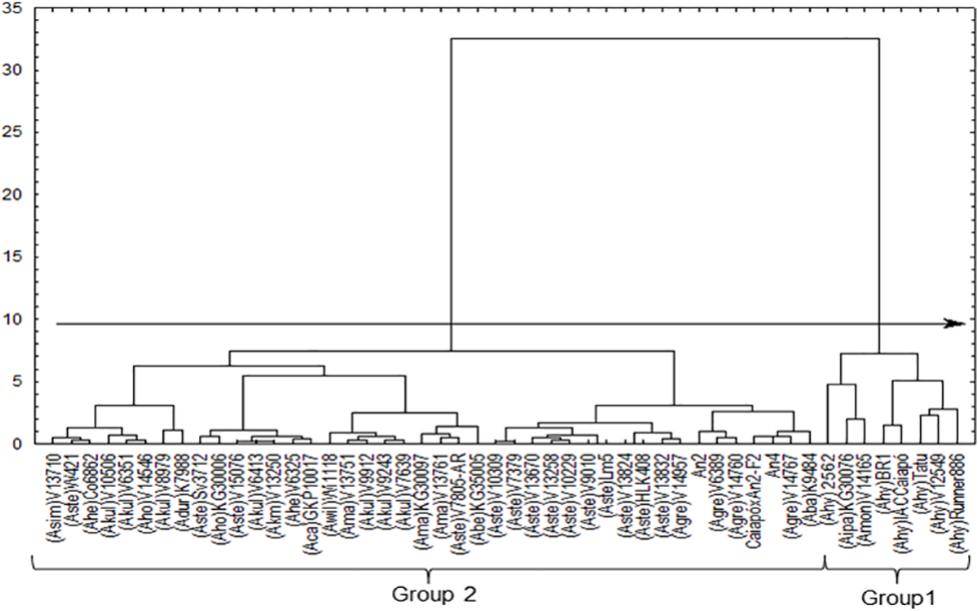
Distribution of wild *Arachis* genotypes and *A*. *hypogaea* controls with respect to resistance to early leaf spot, late leaf spot, rust, and scab in the first year of study. Cut-off point = 10 (arrow) indicates genotype segregation into two groups.

A first GA was conducted with the 50 genotypes (Fig 1). At cut-off point 10 of this dendrogram, genotypes are divided into two groups: Group 1 encompasses all six accessions of *A*. *hypogaea*, *A*. *monticola* (V 14165) and *A*. *ipaënsis* (KG 30076). Interestingly, *A*. *monticola* is a tetraploid species closely related to, and most likely a direct ancestor of, *A*. *hypogaea* [15]. Evidence also suggests that *A*. *ipaënsis* was the B genome species that originated *A*. *hypogaea* [16–18]. Group 2 encompasses all other wild genotypes included in the study, indicating that the majority of wild species are very distinct from cultivated peanut with regards to resistance to evaluated fungal diseases. This finding also suggests that many unexplored genes may be present in these pools that could be introduced into the genome of *A*. *hypogaea*. Another important GA outcome is the grouping of three hybrids (two amphidiploids—An 2 and An 4—and the F_2_ progeny individuals of Caiapó x An 4) in Group 2, as they all kept resistance patterns similar to those of wild species. This finding shows that resistance is maintained after interspecific crossings.

**Fig 2:**
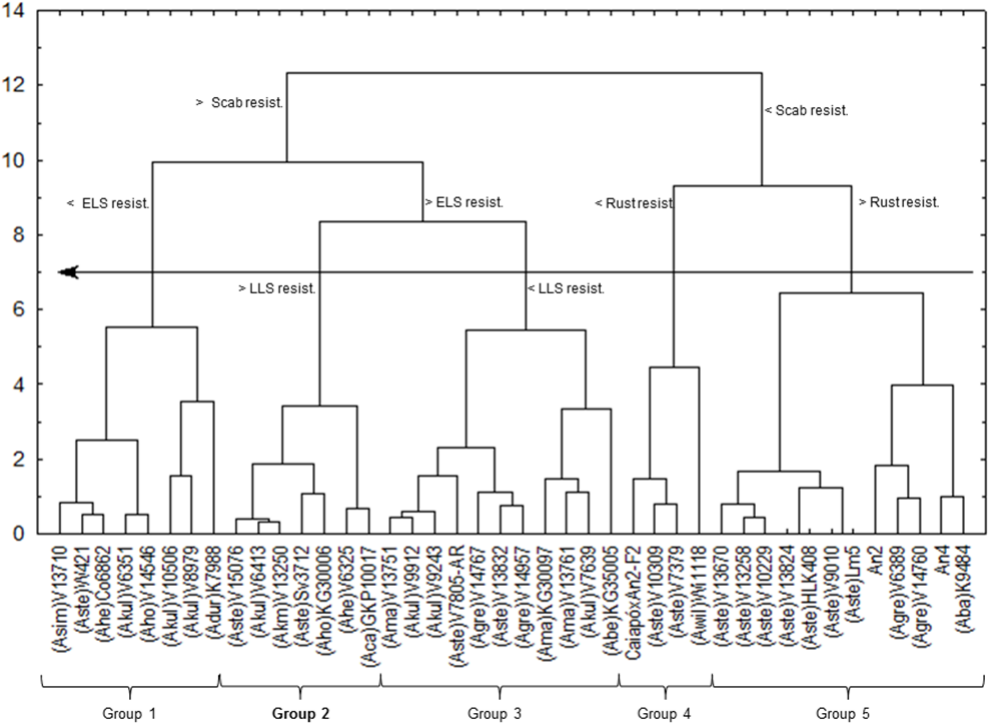
Distribution of wild *Arachis* genotypes according to resistance to late leaf spot (LLS), early leaf spot (ELS), rust and scab, in the first year of study, excluding susceptible groups (accessions of *A*. *hypogaea* and two closely related wild species). Cut-off point = 7 (arrow) indicates genotype segregation into five groups.

If the more susceptible accessions are removed from the analysis, a more detailed picture of wild accession differentiation emerges (Fig 2): a cut-off point of 11 separated accessions according to their resistance to scab, whereas a cut-off point of 7 then discriminated between five genotype groups. The genotypes with least resistance to scab were subdivided as to their resistance to rust. The ones more resistant to scab were further subdivided as to their resistance to early leaf spot. Among the early leaf spot resistant genotypes, another division was possible with regard to resistance to late leaf spot. Therefore, the joint evaluation of four diseases indicates that Group 2 of Fig 2 provides the most resistant accessions and might be the best one for multiple selections. The seven accessions that comprise this group are V 15076 (*A*. *stenosperma*), V 6413 (*A*. *kuhlmannii*), V 13250 (*A*. *kempff-mercadoi*), Sv 3712 (*A*. *stenosperma*), KG 30006 (*A*. *hoehnei*), V 6325 (*A*. *helodes*), and GKP 10017 (*A*. *cardenasii*). All of these accessions are of A genome type, and apparently A genome species are more resistant to fungal diseases than species with other genomes in the *Arachis* section. Validating this observation is difficult due to the smaller number of B genome sensu lato accessions evaluated in this report, which makes it difficult to investigate true variability when compared to the number of A genome accessions.

Another important aspect of resistance is the variability observed among accessions of a single species. Pande and Rao [8] have previously emphasized the importance of evaluating reactions at the individual level. We show that *A*. *stenosperma* accessions are present in every group, whereas *A*. *kuhlmannii* are present in three, *A*. *hoehnei* in two, and *A*. *gregoryi* in two groups. A wider distribution of *A*. *stenosperma* may be a result from a larger number of accessions in this species. If there were more accessions in the other species, we might have observed a similarly ample distribution. As it was observed the wide variability at the species level, research efforts are necessary in the identification of resistances in accessions, not in species.

The two amphidiploids were grouped in Group 5. Amphidiploid An 2 remained very close to one of its progenitors, V 6389 (*A*. *gregoryi*). The other progenitor, V 9401 (*A*. *linearifolia*), was not included in the study due to an insufficient number of seeds. Amphidiploid An 4 resulted from a cross between *A*. *ipaënsis* x *A*. *duranensis* V 14167 followed by artificial polyploidization. The female progenitor fell into Group 1, along with accessions of *A*. *hypogaea*. The *A*. *duranensis* accession was not included in the study for lack of seeds. Interestingly, some ramifications within Group 5 included amphidiploids and other B and K genome species, but no A genome accessions. In another subdivision of Group 5, only A genome accessions were segregated. Non-A genomes were also concentrated in Groups 3 and 4. The F_2_ progeny of IAC Caiapó x An 4, such as *A*. *stenosperma* V 10309, were situated in Group 4, exhibiting partial resistance when compared to wild genotypes.

Four accessions that showed special potential for future studies are the *A*. *magna* accessions V 13751 and KG 30097 and the *A*. *gregoryi* accessions V 14767 and V 14957. While they were not the best in terms of resistance, they belong to the B genome type that is crucial for resistance-gene introgression and pyramidization in *A*. *hypogaea*.

**Fig 3:**
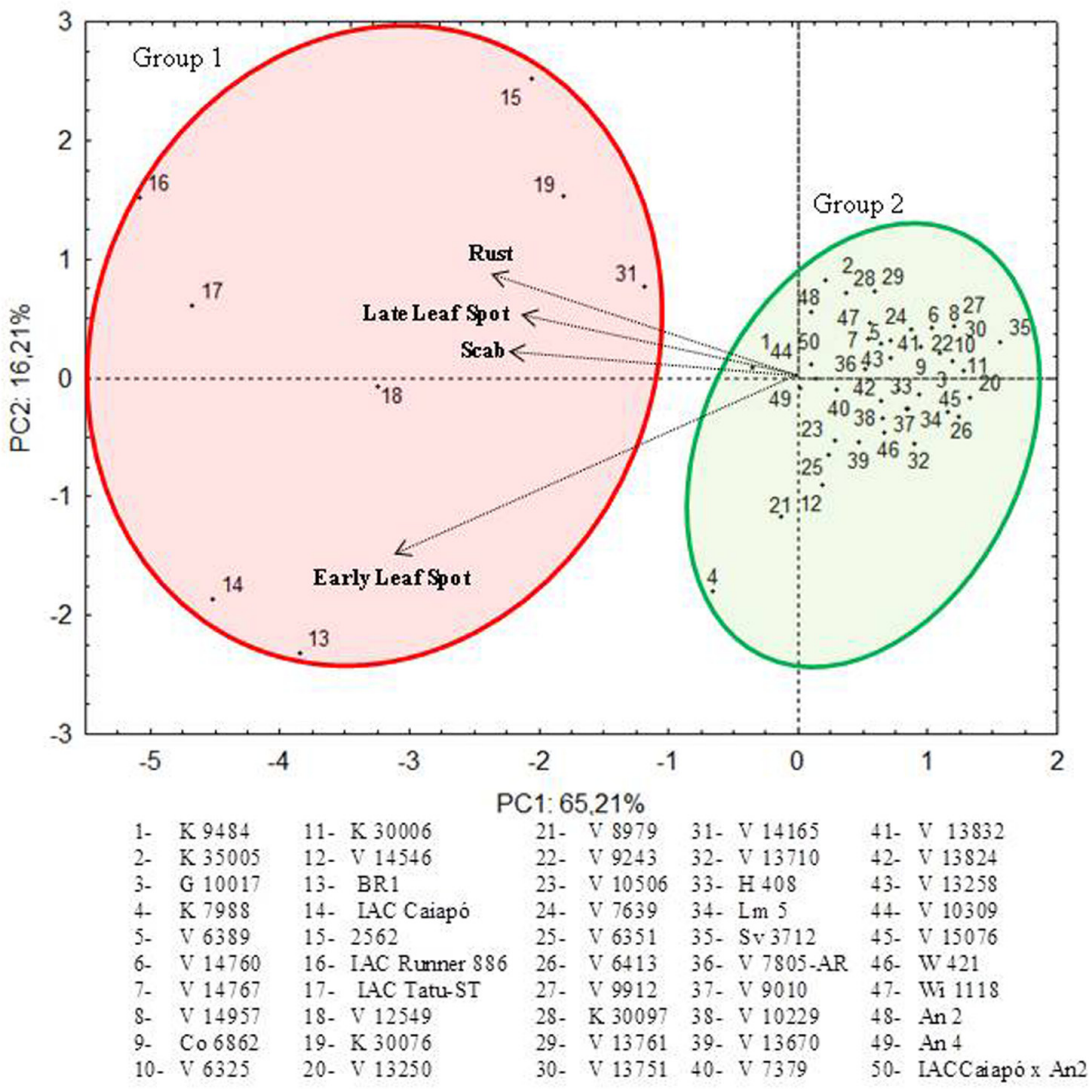
Distribution of wild *Arachis* accessions and *A*. *hypogaea* controls according to their resistance to late leaf spot, early leaf spot, rust and scab in the first year of study. PCA with the two first components explaining 81.41% of variation. Group 1 (red circle) and Group 2 (green circle) include susceptible and resistant accessions respectively.

Similarly to the GA with a cut-off point of 10, a two-group division was observed through PCA (Fig 3), where the two first components explained 81.41% of variation. Again, Group 1 (red circle) was formed by the same eight genotypes as in Fig 1, whereas the other wild accessions were tightly connected in Group 2 (green circle). Arrows point towards accessions, including those of Group 1, which were more susceptible to the diseases evaluated. Some Group 1 genotypes such as IAC-Caiapó and BR-1 were more susceptible to early leaf spot, whereas cultivars IAC-Tatu-ST and IAC-Runner 886 were more strongly associated with late leaf spot. V12549 was more susceptible to scab. Finally, accessions of *A*. *hypogaea* 2562, *A*. *monticola* V14165 and *A*. *ipaënsis* 30076 were more strongly correlated with rust. Accession V14165 was almost equidistant from Group 1 and 2 accessions.

Group 2 encompassed accessions and hybrids that were opposite to the arrows, indicating a trend to multiple resistances of wild genotypes. Again, the two amphidiploids (An 2 and An 4) and the F_2_ progeny individuals of Caiapó x An 4 grouped with wild species. Because Group 2 genotypes were very closely associated, a more refined analysis to define which one would be preferred for genetic improvement required re-running PCA without Group 1 accessions.

**Fig 4:**
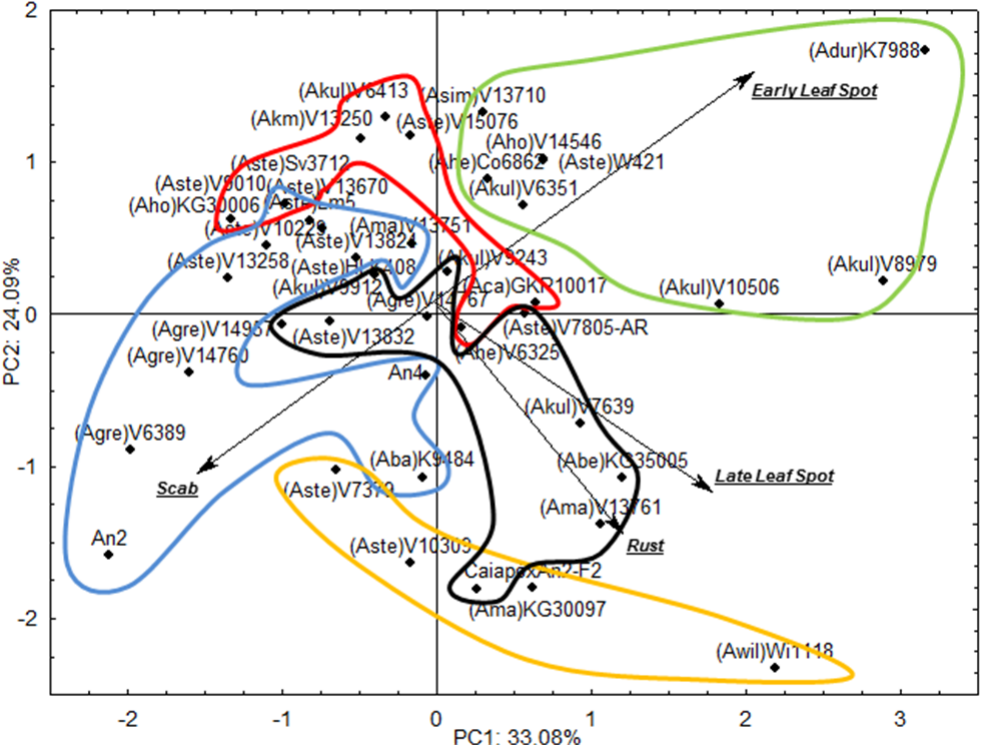
Distribution of wild *Arachis* accessions according to their resistance to late leaf spot, early leaf spot, rust and scab in the first year of study. PCA with the two first components explaining 57.17% of variation. Green, red, black, yellow and blue groups means groups 1, 2, 3, 4 and 5 of Fig 2 respectively.

Fig 4 shows the PCA re-run without Group 1 accessions. The two main components explain 57.17% of variation. When the accessions in Fig 4 were divided into the five GA groups obtained from Fig 2, these groups tended to disperse, with few intersections. Group 1 genotypes, in green, showed less resistance to early leaf spot; and Group 4, in yellow, was the least resistant to rust. Group 3, in black, was more closely associated to late leaf spot and rust; and Group 5, in blue, was in the same direction as the scab arrow, but showing a fair amount of internal variation. For example, Group 5 amphidiploid An 2 had lower resistance to scab whereas Lm 5 was on the border with Group 2, distant from each one of the arrows. In fact, amphidiploids An 2 and An 4 had lower resistance to scab but were more resistant to early leaf spot, rust and late leaf spot. On the other hand, the F_2_ of Caiapó x An 2 showed reduced resistance to rust, late leaf spot and scab, but greater resistance to early leaf spot. An important obstacle to the selection of progenies from interspecific crossings targeting disease resistance is the risk of losing important alleles as a result of backcrossing.

Accessions in Group 2 of Fig 2, in red, were distant from all arrows, and therefore are more likely to have multiple resistances. Overall, PCA validated the GA results.

Table 5 shows average values from the studies conducted in three consecutive years evaluating resistance to fungal diseases in 18 wild accessions and in the cultivar IAC Caiapó. There was a difficulty of selection of the best accessions for the three diseases, justifying again the PCA and grouping analysis utilization. It was also observed that there were differences among years. ANOVA results showed the interaction between accessions x years.

**Table 5:** Average grades of resistance to late leaf spot, early leaf spot and rust of genotypes evaluated during three consecutive years and differences among years averages.

Species/Accessions	Late Leaf Spot	Early Leaf Spot	Rust	Fig 2 Group	Fig 5 Group
*A*. *simpsonii* V 13710	2.22 bcdefg^1^	1.83 b	1.00 b	1	2
*A*. *helodes* Co 6862	2.17 defgh	1.53 bcd	1.03 b	1	2
***A*. *kuhlmannii* V 6413**	1.70 h	1.64 bcd	1.00 b	**2**	**2**
***A*. *stenosperma* V 15076**	1.91 gh	1.46 bcd	1.12 b	**2**	**2**
***A*. *kempff-mercadoi* V 13250**	1.97 fgh	1.44 bcd	1.00 b	**2**	**2**
***A*. *cardenasii* GKP 10017**	2.27 bcdefg	1.27 cd	1.09 b	**2**	**3**
***A*. *helodes* V 6325**	2.18 bcdefg	1.36 cd	1.09 b	**2**	**3**
***A*. *stenosperma* Sv 3712**	2.00 efgh	1.27 d	1.27 b	**2**	**3**
*A*. *gregoryi* V 14767	2.83 b	1.58 bcd	1.03 b	3	1
*A*. *kuhlmannii* V 9912	2.57 bcd	1.31 d	1.00 b	3	1
*A*. *stenosperma* V 13832	2.65 bc	1.51 bcd	1.00 b	3	1
*A*. *stenosperma* V 10309	2.17 cdefg	1.79 bc	1.10 b	4	2
*A*. *stenosperma* V 7379	2.00 fgh	1.64 bcd	1.09 b	4	2
*A*. *stenosperma* V 13670	2.57 bcde	1.43 cd	1.00 b	5	1
*A*. *stenosperma* HLK 408	2.27 bcdefg	1.54 bcd	1.03 b	5	2
*A*. *helodes* Lm5	2.29 bcdefg	1.39 cd	1.24 b	5	3
*A*. *stenosperma* V 9010	2.33 bcdef	1.27 cd	1.00 b	5	3
*A*. *stenosperma* V 13258	2.31 bcdefg	1.42 bcd	1.00 b	5	3
*A*. *hypogaea* IAC Caiapó	6.82 a	5.63 a	5.92 a	*	*
Year 1	3.00 a	1.79 b	1.25 b		
Year 2	2.31 c	1.32 c	1.33 b		
Year 3	2.97 b	2.62 a	2.01 a		

Accessions displaying multiple resistance in bold.

^1.^Distinct letters indicate significant differences among accessions according to Duncan’s test (p<0,05)

* Not included in GA

Of the seven accessions identified as the most resistant (Group 2 of Fig 2), six are shown in bold in Table 5; only accession KG 30006 was not included, because at the time it was not believed to be an A genome species [2]. Additionally, previous tests had failed in crossing and generating fertile amphidiploids from this species. Currently, it is known that this species has the A genome [19], and further work is needed to validate its potential as a male progenitor in interspecific crossings and generation of new amphidiploids.

During GA with all 18 genotypes and the control, only two groups were obtained, because IAC Caiapó was considered susceptible when compared to the wild accessions. Therefore, we removed the control from the analysis to evaluate isolated behavior among the accessions.

**Fig 5:**
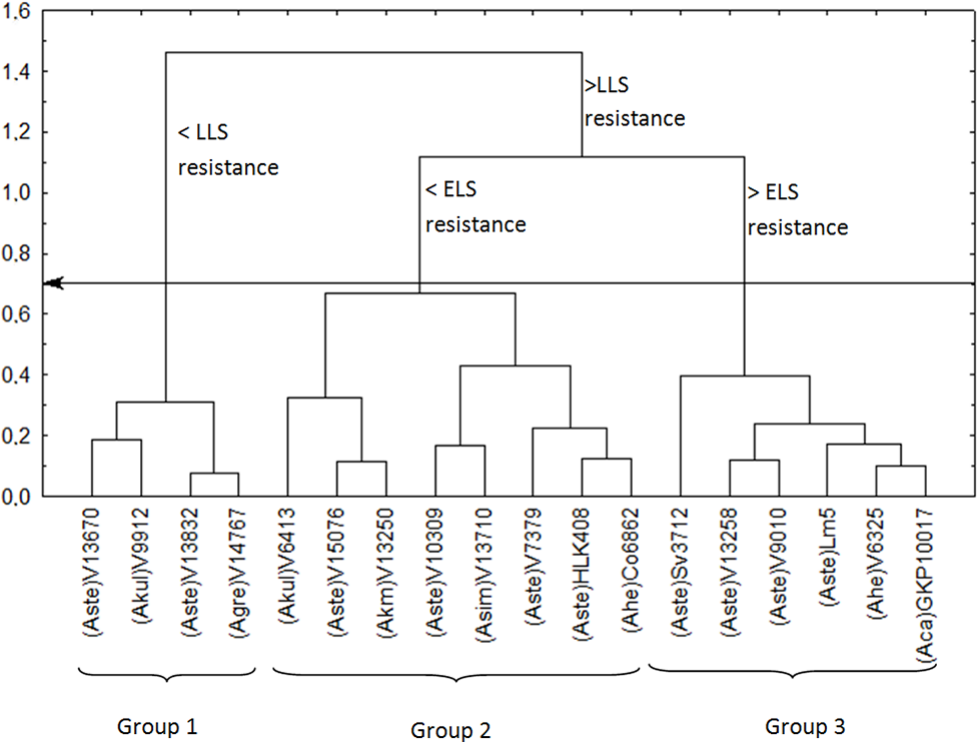
Wild *Arachis* genotypes segregated according to their resistance to late leaf spot (LLS), early leaf spot (ELS), and rust after three years of study, excluding IAC Caiapó control. Cut-off point = 0.7 (arrow) indicates genotype distribution into three groups.

Fig 5 shows GA where a cut-off point of 0.7 forms three groups. All species has A genome, except *A*. *gregoryi* accession V 14767. Rust resistance was not relevant to discriminate between accessions, as they all had low grades, i.e., low infection rates. Comparing data from Table 5 with Fig 5, we may conclude that Group 1 accessions had lower resistance to late leaf spot, whereas Groups 2 and 3 showed greater resistance to this disease. The distinguishing feature between Groups 2 and 3 was that the former included accessions with lower resistance to early leaf spot, compared to the latter.

Therefore, our data suggest that Group 2 accessions (Fig 5) have the greatest potential for use in genetic improvement programs. However, given that late leaf spot is the most important disease in the field, and that the difference in resistance to early leaf spot was small between Groups 2 and 3, both of these groups should be considered in improvement programs. Again, only A genome species were selected, except for one B genome accession, *A*. *gregoryi* V 14767, which segregated to Group 1. V14767 may not be considered the best resistance genotype, but might prove to be an excellent allele donor for gene pyramiding. We must again point out that differences observed among data from Table 5, Figs 2 and 5 result from the fact that, in the first year, we evaluated scab resistance whereas in later years the disease occurred at a very low rate and could not be quantified. Overall, the data show that the best accessions regarding multiple resistance to diseases in this study conditions are V 15076 (*A*. *stenosperma*), V 6413 (*A*. *kuhlmannii*), V 13250 (*A*. *kempff-mercadoi*), Sv 3712 (*A*. *stenosperma*), V 6325 (*A*. *helodes*), GKP 10017 (*A*. *cardenasii*) (Table 5 - bold).

The individual REML analysis (Table 6) of 19 genotypes used in three years assays detected that the environmental variance value was low, allowing the discrimination of genotypes. Based on individual BLUP (Table 6), resistance ranking of each accession was obtained, as well as a general ranking was observed by the sum of all ranks of the three diseases. The highest values are those with the best resistance to the three diseases. Accessions in bold in Table 5 had GR values higher than 32 in Table 6, corroborating the results of Duncan Test, PCA and GA.

**Table 6:** Estimatives of components of variance (Individual REML) and the components of average (Individual BLUP) for the variables resistance to late leaf spot (LLS), early leaf spot (ELS) and rust (R) and 19 genotypes, during three consecutive years.

	Components of average (Individual BLUP)															
Genotype	MP					MC					F					
	Rank	g*	u + g	GG	Na	Rank	g	u + g	GG	Na	Rank	g	u + g	GG	Na	GR
IAC Caiapó	1	4.3741	6.8828	4.3741	6.8828	1	3.5913	5.289	3.5913	5.2890	1	4.3085	5.6253	4.3085	5.6253	3
V 14767	2	0.3123	2.8210	2.3432	4.8519	6	-0.1062	1.5916	0.5880	2.2857	10	-0.2690	1.0478	0.2657	1.5825	18
V 10309	13	-0.3218	2.1869	0.2412	2.7498	3	0.0787	1.7764	1.2507	2.9484	5	-0.2047	1.1121	0.7650	2.0819	21
Lm 5	8	-0.2021	2.3066	0.5586	3.0672	14	-0.2846	1.4131	0.1308	1.8286	3	-0.0633	1.2535	1.4036	2.7205	25
V 13832	3	0.1674	2.6761	1.618	4.1266	7	-0.1292	1.5686	0.4855	2.1833	18	-0.2981	1.0188	0.0167	1.3336	28
V 7379	16	-0.4792	2.0294	0.1156	2.6243	4	-0.0590	1.6387	0.9232	2.6210	8	-0.2101	1.1067	0.3993	1.7162	28
HLK 408	10	-0.2169	2.2918	0.4036	2.9122	8	-0.1435	1.5542	0.4069	2.1046	11	-0.2690	1.0478	0.2170	1.5339	29
V 13710	11	-0.2753	2.2334	0.3419	2.8505	2	0.0821	1.7798	1.8367	3.5344	16	-0.2981	1.0188	0.0561	1.3729	29
Co 6862	14	-0.3290	2.1797	0.2005	2.7091	9	-0.1584	1.5394	0.3441	2.0418	9	-0.2690	1.0478	0.3251	1.6419	32
V 15076	18	-0.5646	1.9440	0.0427	2.5514	10	-0.228	1.4698	0.2869	1.9846	4	-0.1811	1.1357	1.0074	2.3243	32
V 9912	4	0.1119	2.6206	1.2415	3.7501	16	-0.3275	1.3703	0.0745	1.7723	13	-0.2981	1.0188	0.1378	1.4546	33
GKP 10017	9	-0.2160	2.2926	0.4725	2.9812	19	-0.3978	1.2999	0.000	1.6978	6	-0.2101	1.1067	0.6025	1.9193	34
V 6325	12	-0.3032	2.2055	0.2881	2.7968	15	-0.3125	1.3853	0.1013	1.7991	7	-0.2101	1.1067	0.4864	1.8032	34
Sv 3712	15	-0.4775	2.0312	0.1553	2.6639	18	-0.397	1.3008	0.0221	1.7199	2	-0.0343	1.2826	2.1371	3.4539	35
V 13670	5	0.0601	2.5688	1.0052	3.5138	12	-0.2523	1.4454	0.1983	1.8960	19	-0.3011	1.0157	0.0000	1.3168	36
V 13258	7	-0.1959	2.3127	0.6672	3.1759	13	-0.2628	1.435	0.1628	1.8606	17	-0.2981	1.0188	0.0352	1.3521	37
V 9010	6	-0.1594	2.3493	0.8111	3.3197	17	-0.3970	1.3008	0.0468	1.7445	15	-0.2981	1.0188	0.0797	1.3965	38
V 6413	19	-0.7694	1.7392	0.0000	2.5087	5	-0.0590	1.6387	0.7268	2.4246	14	-0.2981	1.0188	0.1067	1.4235	38
V 13250	17	-0.5158	1.9929	0.0785	2.5871	11	-0.2371	1.4607	0.2392	1.9370	12	-0.2981	1.0188	0.1741	1.491	40
Components Of Variance (Individual REML)	V_g_ = 1.2444					V_g_ = 0.8351						V_g_ = 1.1322				
	Vperm = 0.0627					V_perm_ = 0.0751						V_perm_ = 0.0445				
	V_e_ = 0.5281					V_e_ = 0.6229						V_e_ = 0.3777				
	V_f_ = 1.8352					V_f_ = 1.5331						V_f_ = 1.5545				
	h^2^_g_ = 0.6781 +- 0.1596					h^2^_g_ = 0.5447 +- 0.1427						h^2^_g_ = 0.7284 +- 0.1678				
	r = 0.7122 +- 0.1636					r = 0.5937 +- 0.1490						r = 0.7570 +- 0.1710				
	c^2^_perm_ = 0.03414					c^2^_perm_ = 0.04897						c^2^_perm_ = 0.0286				
	h^2^_mg_ = 0.8647					h^2^_mg_ = 0.7835						h^2^_mg_ = 0.8907				
	GA = 2.5087					GA = 1.6978						GA = 1.3168				

* g: genotypic efect, u + g: genotypic average, GG: genetic gain, Na = new average, GR: general rank, V_g_: genotypic variance, V_perm_: variance of the permanent enviromental effects, V_e_: residual variance, V_f_: phenotypic variance, h^2^_g_ = plot heritability in broad sense, r: plot repeatability, c^2^_perm_ = enviroment determination coefficient, h^2^_mg_: genotype average heritability, GA- General Average

Variance analysis showed that *A*. *kuhlmannii* (V 6413) had the lowest average degrees of observation of late leaf spot, whereas *A*. *stenosperma* (Sv 3712) and *A*. *kuhlmannii* (V 9912) had the lowest incidence (lowest grade) of early leaf spot. All wild genotypes showed resistance to rust at the natural inoculum pressure used (Table 5).

Fávero *et al*. [9] utilized detached leaves to show that *Arachis hypogaea* and *Arachis monticola* were susceptible to late leaf spot, early leaf spot and rust, as we reproduced here in the field. Similarly, our results agree with those of Fávero *et al*. [9] with regards to V9243 susceptibility to late leaf spot, and Wi 1118 and V13824 susceptibility to rust. However, in contrast to that previous work, we show that in three years of field evaluation the Sv 3712 accession was resistant to rust. Yet another distinct new finding of our study is the susceptibility of *A*. *batizocoi* to scab; Fávero *et al*. [9] found this accession to be highly resistant for late and early leaf spots but did not test it for scab.

Pande and Rao [8] also identified late leaf spot resistance in an *A*. *hoehnei* accession collected at a site near the collection site of the species used in our study, and they reported the same result for their KG 30006 accession from the same region. In both studies, *A*. *monticola* accessions were susceptible to late leaf spot and rust.

## Conclusions

We have found accessions with greater resistance to disease than *A*. *cardenasii*. The most promising accessions with multiple resistance to late leaf spot, early leaf spot, rust and scab in our study conditions were V 15076 (*A*. *stenosperma*), V 6413 (*A*. *kuhlmannii*), V 13250 (*A*. *kempff-mercadoi*), Sv 3712 (*A*. *stenosperma*), KG 30006 (*A*. *hoehnei*), V 6325 (*A*. *helodes*) and GKP 10017 (*A*. *cardenasii*). Amphidiploids and *A*. *hypogaea* x amphidiploid hybrids behaved similarly to wild species. Four accessions that should be further evaluated are the *A*. *magna* accessions V 13751 and KG 30097 and the *A*. *gregoryi* accessions V 14767 and V 14957. Although they did not show specifically high resistance, they belong to the B genome type that is crucial to resistance gene introgression and pyramiding in *A*. *hypogaea*.

## Supporting Information

S1 Table Raw data of 50 *Arachis* genotypes evaluated for resistance to late leaf spot, early leaf spot, rust and scab in field assays. (DOC)
