# Supplementary material for: Identification of Fungus Resistant Wild Accessions and Interspecific Hybrids of the Genus Arachis
Source: PLoS One. 2015 Jun 19;10(6):e0128811. doi: 10.1371/journal.pone.0128811 (PMC4474867; doi:10.1371/journal.pone.0128811)
Supplement: S1 Table — (DOC) [file pone.0128811.s001.doc]

**S1 Table. Raw data of 50 *Arachis* genotypes evaluated for resistance to late leaf spot, early leaf spot, rust and scab in field assays**.

| Cultivar | Rep | 1st year | | | | 2nd year | | | 3rd year | | |
| --- | --- | --- | --- | --- | --- | --- | --- | --- | --- | --- | --- |
|  |  | LLS | ELS | S | R | LLS | ELS | R | LLS | ELS | R |
| (Aca) GKP 10017 | 1 | 3,00 | 2,00 | 1,00 | 1,00 | 2,00 | 1,00 | 1,00 | 1,67 | 1,33 | 1,00 |
| (Aca) GKP 10017 | 2 | 3,00 | 2,00 | 1,00 | 1,00 | 2,00 | 1,00 | 1,00 | 2,67 | 1,00 | 1,00 |
| (Aca) GKP 10017 | 3 | 2,00 | 1,00 | 1,00 | 2,00 | 1,67 | 1,00 | 1,00 | 2,00 | 1,33 | 1,00 |
| (Aca) GKP 10017 | 4 | . | . | . | . | 2,00 | 1,33 | 1,00 | 3,00 | 1,00 | 1,00 |
| (Ahy) IAC Caiapó | 1 | 7,50 | 5,75 | 2,25 | 4,50 | 3,56 | 2,33 | 2,11 | 9,00 | 9,00 | 9,00 |
| (Ahy) IAC Caiapó | 2 | 7,75 | 5,00 | 2,25 | 5,00 | 3,78 | 3,56 | 2,78 | 8,50 | 8,50 | 8,50 |
| (Ahy) IAC Caiapó | 3 | 7,75 | 5,75 | 2,75 | 3,00 | 6,00 | 3,50 | 4,67 | 8,50 | 8,50 | 8,50 |
| (Ahy) IAC Caiapó | 4 | 7,00 | 4,25 | 2,50 | . | 6,83 | 1,83 | 6,83 | 8,50 | 8,50 | 8,50 |
| (Ahe) V 6325 | 1 | 2,00 | 2,00 | 1,00 | 2,00 | 1,67 | 1,00 | 1,00 | 2,00 | 2,00 | 1,00 |
| (Ahe) V 6325 | 2 | 3,00 | 1,00 | 1,00 | 1,00 | 2,00 | 1,00 | 1,00 | 2,00 | 1,67 | 1,00 |
| (Ahe) V 6325 | 3 | 2,00 | 1,00 | 1,00 | 1,00 | 2,00 | 1,00 | 1,00 | 2,67 | 1,00 | 1,00 |
| (Ahe) V 6325 | 4 | . | . | . | . | 1,67 | 1,00 | 1,00 | 3,00 | 2,33 | 1,00 |
| (Ahe) Co 6862 | 1 | 3,00 | 2,00 | 1,00 | 1,00 | 2,00 | 1,00 | 1,00 | 1,67 | 1,00 | 1,00 |
| (Ahe) Co 6862 | 2 | 3,00 | 2,00 | 1,00 | 1,00 | 1,33 | 1,00 | 1,00 | 2,00 | 2,00 | 1,33 |
| (Ahe) Co 6862 | 3 | 4,00 | 2,00 | 1,00 | 1,00 | 1,67 | 1,00 | 1,00 | 2,00 | 1,67 | 1,00 |
| (Ahe) Co 6862 | 4 | 3,00 | 1,00 | 1,00 | . | 1,00 | 1,67 | 1,00 | 1,33 | 2,00 | 1,00 |
| (Agre) V 14767 | 1 | 5,00 | 2,00 | 1,00 | 1,00 | 1,67 | 1,67 | 1,00 | 2,33 | 1,67 | 1,00 |
| (Agre) V 14767 | 2 | 5,00 | 2,00 | 1,00 | 1,00 | 2,00 | 1,00 | 1,00 | 2,00 | 1,00 | 1,00 |
| (Agre) V 14767 | 3 | 2,00 | 1,00 | 1,00 | 1,00 | 2,00 | 1,33 | 1,00 | 3,00 | 3,00 | 1,00 |
| (Agre) V 14767 | 4 | 4,00 | 1,00 | 2,00 | . | 3,00 | 1,67 | 1,00 | 2,00 | 1,67 | 1,33 |
| (Akm) V 13250 | 1 | 2,00 | 2,00 | 1,00 | 1,00 | 1,33 | 1,00 | 1,00 | 1,67 | 1,00 | 1,00 |
| (Akm) V 13250 | 2 | 2,00 | 2,00 | 1,00 | 1,00 | 2,00 | 2,00 | 1,00 | 2,00 | 1,33 | 1,00 |
| (Akm) V 13250 | 3 | 2,00 | 1,00 | 1,00 | 1,00 | 1,67 | 1,00 | 1,00 | 2,33 | 1,33 | 1,00 |
| (Akm) V 13250 | 4 | 2,00 | 1,00 | 1,00 | . | 2,00 | 2,00 | 1,00 | 2,67 | 1,67 | 1,00 |
| (Akul) V 9912 | 1 | 4,00 | 1,00 | 1,00 | 1,00 | 2,00 | 1,00 | 1,00 | 2,67 | 1,33 | 1,00 |
| (Akul) V 9912 | 2 | 3,00 | 1,00 | 1,00 | 1,00 | 2,50 | 1,17 | 1,00 | 2,00 | 1,00 | 1,00 |
| (Akul) V 9912 | 3 | 3,00 | 1,00 | 1,00 | 1,00 | 2,00 | 1,00 | 1,00 | 2,00 | 3,00 | 1,00 |
| (Akul) V 9912 | 4 | 3,00 | 1,00 | 1,00 | . | 3,00 | 1,00 | 1,00 | 2,33 | 2,67 | 1,00 |
| (Akul) V 6413 | 1 | 2,00 | 2,00 | 1,00 | 1,00 | 2,00 | 1,00 | 1,00 | 2,00 | 1,33 | 1,00 |
| (Akul) V 6413 | 2 | 2,00 | 2,00 | 1,00 | 1,00 | 1,00 | 1,00 | 1,00 | 2,00 | 1,33 | 1,00 |
| (Akul) V 6413 | 3 | 2,00 | 1,00 | 1,00 | 1,00 | 1,67 | 1,67 | 1,00 | 1,33 | 2,00 | 1,00 |
| (Akul) V 6413 | 4 | . | . | . | . | 1,33 | 1,67 | 1,00 | 1,33 | 3,00 | 1,00 |
| (Aste) V 15076 | 1 | 3,00 | 2,00 | 1,00 | 1,00 | 1,00 | 1,00 | 1,00 | 1,67 | 1,67 | 1,00 |
| (Aste) V 15076 | 2 | 2,00 | 2,00 | 1,00 | 1,00 | 2,00 | 1,00 | 1,00 | 2,00 | 1,33 | 1,33 |
| (Aste) V 15076 | 3 | 2,00 | 1,00 | 1,00 | 1,00 | 1,67 | 1,00 | 1,00 | 1,00 | 2,33 | 2,00 |
| (Aste) V 15076 | 4 | . | . | . | . | 2,00 | 1,00 | 1,00 | 2,67 | 1,67 | 1,00 |
| (Aste) Lm5 | 1 | 3,00 | 2,00 | 1,00 | 1,00 | 3,00 | 1,00 | 1,00 | 2,00 | 3,00 | 1,33 |
| (Aste) Lm5 | 2 | 2,00 | 2,00 | 1,00 | 1,00 | 2,00 | 1,00 | 1,00 | 2,67 | 1,00 | 2,67 |
| (Aste) Lm5 | 3 | 2,00 | 1,00 | 2,00 | 1,00 | 1,33 | 1,00 | 1,00 | 2,67 | 1,33 | 1,00 |
| (Aste) Lm5 | 4 | . | . | . | . | 2,50 | 1,00 | 1,00 | 2,00 | 1,00 | 1,67 |
| (Aste) HLK 408 | 1 | 5,00 | 2,00 | 1,00 | 1,00 | 1,67 | 1,00 | 1,33 | 2,33 | 1,00 | 1,00 |
| (Aste) HLK 408 | 2 | 2,00 | 2,00 | 1,00 | 1,00 | 1,67 | 1,00 | 1,00 | 2,00 | 2,00 | 1,00 |
| (Aste) HLK 408 | 3 | 2,00 | 1,00 | 2,00 | 1,00 | 1,33 | 1,00 | 1,00 | 2,67 | 2,00 | 1,00 |
| (Aste) HLK 408 | 4 | . | . | . | . | 2,33 | 1,00 | 1,00 | 2,00 | 3,00 | 1,00 |
| (Aste) V 10229 | 4 | 3,00 | 1,00 | 2,00 | . | 2,00 | 1,00 | 1,00 | 3,00 | 1,00 | 1,00 |
| (Aste) V 9010 | 1 | 3,00 | 2,00 | 1,00 | 1,00 | 2,00 | 1,00 | 1,00 | 2,33 | 1,67 | 1,00 |
| (Aste) V 9010 | 2 | 2,00 | 2,00 | 1,00 | 1,00 | 2,00 | 1,00 | 1,00 | 3,00 | 1,00 | 1,00 |
| (Aste) V 9010 | 3 | 2,00 | 1,00 | 2,00 | 1,00 | 2,00 | 1,33 | 1,00 | 2,33 | 1,00 | 1,00 |
| (Aste) V 9010 | 4 | . | . | . | . | 2,00 | 1,67 | 1,00 | 1,33 | 1,67 | 1,00 |
| (Asim) V 13710 | 1 | 2,00 | 2,00 | 1,00 | 1,00 | 2,00 | 1,00 | 1,00 | 2,00 | 2,67 | 1,00 |
| (Asim) V 13710 | 2 | 3,00 | 2,00 | 1,00 | 1,00 | 2,00 | 1,00 | 1,00 | 3,00 | 2,33 | 1,00 |
| (Asim) V 13710 | 3 | 3,00 | 2,00 | 1,00 | 1,00 | 1,67 | 1,00 | 1,00 | 2,33 | 2,33 | 1,00 |
| (Asim) V 13710 | 4 | . | . | . | . | 2,00 | 1,00 | 1,00 | 2,33 | 2,33 | 1,00 |
| (Aste) V 13258 | 1 | 3,00 | 2,00 | 1,00 | 1,00 | 2,00 | 1,00 | 1,00 | 2,00 | 2,00 | 1,00 |
| (Aste) V 13258 | 2 | 2,00 | 2,00 | 1,00 | 1,00 | 1,67 | 1,00 | 1,00 | 3,00 | 1,67 | 1,00 |
| (Aste) V 13258 | 3 | 2,00 | 1,00 | 2,00 | 1,00 | 2,33 | 1,00 | 1,00 | 3,33 | 1,00 | 1,00 |
| (Aste) V 13824 | 3 | 3,00 | 2,00 | 2,00 | 1,00 | 2,00 | 1,00 | 1,00 | 2,00 | 2,00 | 1,00 |
| (Aste) V 13670 | 1 | 2,00 | 1,00 | 2,00 | 1,00 | 2,00 | 1,00 | 1,00 | 3,00 | 1,00 | 1,00 |
| (Aste) V 13670 | 2 | 3,00 | 3,00 | 1,00 | 1,00 | 3,00 | 1,00 | 1,00 | 1,67 | 2,33 | 1,00 |
| (Aste) V 13670 | 3 | . | . | . | . | 4,00 | 1,00 | 1,00 | 3,00 | 1,00 | 1,00 |
| (Aste) V 13670 | 4 | . | . | . | . | 1,67 | 1,00 | 1,00 | 2,00 | 2,00 | 1,00 |
| (Aste) Sv 3712 | 1 | 2,00 | 1,00 | 1,00 | 1,00 | 2,00 | 1,00 | 1,00 | 2,00 | 1,00 | 2,67 |
| (Aste) Sv 3712 | 2 | 2,00 | 1,00 | 1,00 | 1,00 | 1,67 | 1,00 | 1,00 | 2,00 | 2,00 | 1,00 |
| (Aste) Sv 3712 | 3 | 2,00 | 1,00 | 1,00 | 1,00 | 2,00 | 1,00 | 1,00 | 2,67 | 2,00 | 2,33 |
| (Aste) Sv 3712 | 4 | . | . | . | . | 2,75 | 1,50 | 1,00 | 2,00 | 2,67 | 1,00 |
| (Aste) V 13832 | 1 | 3,00 | 1,00 | 1,00 | 1,00 | 2,33 | 1,00 | 1,00 | 2,00 | 2,33 | 1,00 |
| (Aste) V 13832 | 2 | 3,00 | 1,00 | 1,00 | 1,00 | 2,33 | 1,00 | 1,00 | 3,00 | 2,00 | 1,00 |
| (Aste) V 13832 | 3 | 4,00 | 2,00 | 2,00 | 1,00 | 2,33 | 1,00 | 1,00 | 2,67 | 1,67 | 1,00 |
| (Aste) V 13832 | 4 | . | . | . | . | 2,00 | 1,33 | 1,00 | 1,33 | 2,00 | 1,00 |
| (Aste) V 7379 | 1 | 4,00 | 2,00 | 2,00 | 2,00 | 1,00 | 1,00 | 1,00 | 2,00 | 2,00 | 1,00 |
| (Aste) V 7379 | 2 | 2,00 | 2,00 | 1,00 | 1,00 | 1,33 | 1,00 | 1,00 | 2,33 | 1,67 | 1,00 |
| (Aste) V 7379 | 3 | 2,00 | 1,00 | 2,00 | 1,00 | 2,00 | 1,00 | 1,00 | 2,00 | 3,00 | 1,00 |
| (Aste) V 7379 | 4 | . | . | . | . | 2,00 | 1,00 | 1,00 | 2,00 | 2,00 | 1,00 |
| (Aste) V 10309 | 1 | 3,00 | 2,00 | 2,00 | 2,00 | 2,00 | 1,00 | 1,00 | 1,00 | 2,67 | 1,00 |
| (Aste) V 10309 | 2 | 3,00 | 2,00 | 1,00 | 1,00 | 2,00 | 1,00 | 1,00 | 2,00 | 2,33 | 1,00 |
| (Aste) V 10309 | 3 | . | 1,00 | 2,00 | . | 2,67 | 1,67 | 1,00 | 2,00 | 3,00 | 1,00 |
| (Ahy) IAC Runner 886 | 1 | 9,00 | 1,00 | . | . |  |  |  |  |  |  |
| (Ahy) IAC Runner 886 | 2 | 7,75 | 1,00 | 3,00 | 8,00 |  |  |  |  |  |  |
| (Ahy) IAC Runner 886 | 3 | 9,00 | . | 2,00 | . |  |  |  |  |  |  |
| (Ahy) IAC Runner 886 | 4 | 9,00 | 7,00 | 2,00 | . |  |  |  |  |  |  |
| (Aba) K 9484 | 1 | 5,00 | 2,00 | 1,00 | 1,00 |  |  |  |  |  |  |
| (Aba) K 9484 | 2 | 4,00 | 1,00 | 3,00 | 1,00 |  |  |  |  |  |  |
| (Aba) K 9484 | 3 | 7,00 | 2,00 | 1,00 | 1,00 |  |  |  |  |  |  |
| (Abe) KG 35005 | 1 | 5,00 | 1,00 | 1,00 | 1,00 |  |  |  |  |  |  |
| (Abe) KG 35005 | 2 | 8,00 | 1,00 | 1,00 | 1,00 |  |  |  |  |  |  |
| (Abe) KG 35005 | 3 | 7,00 | 1,00 | 1,00 | 1,00 |  |  |  |  |  |  |
| (Abe) KG 35005 | 4 | 7,00 | 1,00 | 1,00 | . |  |  |  |  |  |  |
| (Akul) V 7639 | 1 | 4,00 | 1,00 | 1,00 | 1,00 |  |  |  |  |  |  |
| (Akul) V 7639 | 2 | 4,00 | 2,00 | 1,00 | 1,00 |  |  |  |  |  |  |
| (Akul) V 7639 | 3 | 4,00 | 1,00 | 1,00 | 2,00 |  |  |  |  |  |  |
| (Adur) K 7988 | 1 | 6,00 | 5,00 | 1,00 | 1,00 |  |  |  |  |  |  |
| (Adur) K 7988 | 2 | 6,00 | 4,00 | 1,00 | 1,00 |  |  |  |  |  |  |
| (Adur) K 7988 | 3 | 4,00 | 2,00 | 1,00 | 1,00 |  |  |  |  |  |  |
| (Agre) V 14957 | 1 | 3,00 | 1,00 | 1,00 | 1,00 |  |  |  |  |  |  |
| (Agre) V 14957 | 2 | 2,00 | 1,00 | 1,00 | 1,00 |  |  |  |  |  |  |
| (Agre) V 14957 | 3 | 3,00 | 1,00 | 1,00 | 1,00 |  |  |  |  |  |  |
| (Agre) V 14957 | 4 | 4,00 | 1,00 | 2,00 | . |  |  |  |  |  |  |
| (Agre) V 14760 | 1 | 2,00 | 1,00 | 2,00 | 1,00 |  |  |  |  |  |  |
| (Agre) V 14760 | 2 | 3,00 | 1,00 | 1,00 | 1,00 |  |  |  |  |  |  |
| (Agre) V 14760 | 3 | 3,00 | 1,00 | 2,00 | 1,00 |  |  |  |  |  |  |
| (Agre) V 14760 | 4 | 3,00 | 1,00 | 1,00 | . |  |  |  |  |  |  |
| (Aho) KG 30006 | 1 | 1,00 | 1,00 | 1,00 | 1,00 |  |  |  |  |  |  |
| (Aho) KG 30006 | 2 | 2,00 | 2,00 | 1,00 | 1,00 |  |  |  |  |  |  |
| (Aho) KG 30006 | 3 | 2,00 | 1,00 | 1,00 | 1,00 |  |  |  |  |  |  |
| (Aho) KG 30006 | 4 | 2,00 | 1,00 | 2,00 | . |  |  |  |  |  |  |
| (Aho) V 14546 | 1 | 3,00 | 3,00 | 1,00 | 1,00 |  |  |  |  |  |  |
| (Aho) V 14546 | 2 | 3,00 | 2,00 | 1,00 | 1,00 |  |  |  |  |  |  |
| (Aho) V 14546 | 3 | 4,00 | 4,00 | 1,00 | 1,00 |  |  |  |  |  |  |
| (Aho) V 14546 | 4 | 4,00 | 1,00 | 2,00 | . |  |  |  |  |  |  |
| (A.ste) V 7805-AR | 1 | 5,00 | 2,00 | 1,00 | 1,00 |  |  |  |  |  |  |
| (A.ste) V 7805-AR | 2 | 5,00 | 1,00 | 1,00 | 1,00 |  |  |  |  |  |  |
| (A.ste) V 7805-AR | 3 | 4,00 | 1,00 | 1,00 | 1,00 |  |  |  |  |  |  |
| (Agre) V 6389 | 1 | 2,00 | 1,00 | 1,00 | 1,00 |  |  |  |  |  |  |
| (Agre) V 6389 | 2 | 2,00 | 1,00 | 1,00 | 1,00 |  |  |  |  |  |  |
| (Agre) V 6389 | 3 | 4,00 | 1,00 | 3,00 | 1,00 |  |  |  |  |  |  |
| (Agre) V 6389 | 4 | 4,00 | 1,00 | 2,00 | . |  |  |  |  |  |  |
| (Amon) V 14165 | 1 | 5,00 | 1,00 | 3,00 | 4,00 |  |  |  |  |  |  |
| (Amon) V 14165 | 2 | 5,00 | 2,00 | 1,00 | 1,00 |  |  |  |  |  |  |
| (Amon) V 14165 | 3 | 6,00 | 1,00 | 3,00 | 1,00 |  |  |  |  |  |  |
| (Ahy) V 12549 | 1 | 7,00 | 1,00 | 3,00 | 6,00 |  |  |  |  |  |  |
| (Ahy) V 12549 | 2 | 6,00 | 4,00 | 1,00 | 5,00 |  |  |  |  |  |  |
| (Ahy) V 12549 | 3 | 4,00 | 5,00 | 3,00 | 4,00 |  |  |  |  |  |  |
| (Ahy) V 12549 | 4 | 3,00 | 3,00 | 3,00 | . |  |  |  |  |  |  |
| (Ahy) IAC Tatu-ST | 1 | 9,00 | 6,00 | 4,00 | 5,00 |  |  |  |  |  |  |
| (Ahy) IAC Tatu-ST | 2 | 9,00 | 2,00 | . | . |  |  |  |  |  |  |
| (Ahy) IAC Tatu-ST | 3 | 9,00 | . | . | . |  |  |  |  |  |  |
| (Ahy) IAC Tatu-ST | 4 | 8,00 | 1,00 | 2,00 | . |  |  |  |  |  |  |
| (Ahy) BR1 | 1 | 8,00 | 7,00 | 4,00 | 3,00 |  |  |  |  |  |  |
| (Ahy) BR1 | 2 | 9,00 | . | . | . |  |  |  |  |  |  |
| (Ahy) BR1 | 3 | 7,00 | 3,00 | 1,00 | 1,00 |  |  |  |  |  |  |
| (Aipa) KG 30076 | 1 | 5,00 | 2,00 | 2,00 | 5,00 |  |  |  |  |  |  |
| (Aipa) KG 30076 | 2 | 4,00 | 1,00 | 5,00 | 5,00 |  |  |  |  |  |  |
| (Aipa) KG 30076 | 3 | 3,00 | 1,00 | 2,00 | 4,00 |  |  |  |  |  |  |
| (Aipa) KG 30076 | 4 | 3,00 | 1,00 | 1,00 | . |  |  |  |  |  |  |
| (Akul) V 9243 | 1 | 3,00 | 1,00 | 1,00 | 1,00 |  |  |  |  |  |  |
| (Akul) V 9243 | 2 | 3,00 | 1,00 | 1,00 | 1,00 |  |  |  |  |  |  |
| (Akul) V 9243 | 3 | 4,00 | 2,00 | 1,00 | 1,00 |  |  |  |  |  |  |
| (Akul) V 9243 | 4 | 5,00 | 1,00 | 1,00 | . |  |  |  |  |  |  |
| (Awil) Wi 1118 | 1 | 4,00 | 2,00 | 1,00 | 3,00 |  |  |  |  |  |  |
| (Awil) Wi 1118 | 2 | 3,00 | 1,00 | 1,00 | 1,00 |  |  |  |  |  |  |
| An 4 | 1 | 4,00 | 2,00 | 1,00 | 1,00 |  |  |  |  |  |  |
| An 4 | 2 | 5,00 | 2,00 | 1,00 | 1,00 |  |  |  |  |  |  |
| An 4 | 3 | 5,00 | 2,00 | 1,00 | 1,00 |  |  |  |  |  |  |
| An 4 | 4 | 4,00 | 1,00 | 3,00 | . |  |  |  |  |  |  |
| (Ahyp) 2562 | 1 | 9,00 | . | . | . |  |  |  |  |  |  |
| (Ahyp) 2562 | 2 | 9,00 | . | . | 5,50 |  |  |  |  |  |  |
| (Akul) V 8979 | 1 | 5,00 | 3,00 | 1,00 | 2,00 |  |  |  |  |  |  |
| (Akul) V 8979 | 2 | 3,00 | 3,00 | 1,00 | 1,00 |  |  |  |  |  |  |
| (Akul) V 10506 | 1 | 5,00 | 3,00 | 1,00 | 2,00 |  |  |  |  |  |  |
| (Akul) V 10506 | 2 | 3,00 | 2,00 | 1,00 | 1,00 |  |  |  |  |  |  |
| (Akul) V 10506 | 3 | 4,00 | 2,00 | 1,00 | 1,00 |  |  |  |  |  |  |
| (Akul) V 10506 | 4 | 4,00 | 2,00 | 1,00 | . |  |  |  |  |  |  |
| (Akul) V 6351 | 1 | 3,00 | 2,00 | 1,00 | 1,00 |  |  |  |  |  |  |
| (Akul) V 6351 | 2 | 4,00 | 3,00 | 1,00 | 1,00 |  |  |  |  |  |  |
| (Akul) V 6351 | 3 | 3,00 | 2,00 | 1,00 | 1,00 |  |  |  |  |  |  |
| (Akul) V 6351 | 4 | 5,00 | 2,00 | 2,00 | . |  |  |  |  |  |  |
| (Ama) V 13751 | 1 | 6,00 | 1,00 | 1,00 | 1,00 |  |  |  |  |  |  |
| (Ama) V 13751 | 2 | 3,00 | 2,00 | 1,00 | 1,00 |  |  |  |  |  |  |
| (Ama) V 13751 | 3 | 2,00 | 1,00 | 1,00 | 1,00 |  |  |  |  |  |  |
| (Ama) V 13751 | 4 | 2,00 | 1,00 | 1,00 | . |  |  |  |  |  |  |
| (Ama) V 13761 | 1 | 6,00 | 1,00 | 1,00 | 2,00 |  |  |  |  |  |  |
| (Ama) V 13761 | 2 | 6,00 | 1,00 | 1,00 | 1,00 |  |  |  |  |  |  |
| (Ama) V 13761 | 3 | 3,00 | 1,00 | 1,00 | 1,00 |  |  |  |  |  |  |
| (Ama) KG 30097 | 1 | 6,00 | 1,00 | 2,00 | 2,00 |  |  |  |  |  |  |
| (Ama) KG 30097 | 2 | 6,00 | 1,00 | 1,00 | 1,00 |  |  |  |  |  |  |
| (Ama) KG 30097 | 3 | 2,00 | 1,00 | 1,00 | 1,00 |  |  |  |  |  |  |
| (Aste) W 421 | 1 | 5,00 | 2,00 | 1,00 | 1,00 |  |  |  |  |  |  |
| (Aste) W 421 | 2 | 4,00 | 2,00 | 1,00 | 1,00 |  |  |  |  |  |  |
| (Aste) W 421 | 3 | 3,00 | 2,00 | 1,00 | 1,00 |  |  |  |  |  |  |
| (Aste) W 421 | 4 | 2,00 | . | 1,00 | . |  |  |  |  |  |  |
| (Aste) V 10229 | 1 | 2,00 | 3,00 | 1,00 | 1,00 |  |  |  |  |  |  |
| (Aste) V 10229 | 2 | 2,00 | 2,00 | 1,00 | 1,00 |  |  |  |  |  |  |
| (Aste) V 10229 | 3 | 2,00 | 1,00 | 2,00 | 1,00 |  |  |  |  |  |  |
| (Aste) V 13258 | 4 | 2,00 | 1,00 | 2,00 | . |  |  |  |  |  |  |
| (Aste) V 13824 | 1 | 3,00 | 1,00 | 1,00 | 1,00 |  |  |  |  |  |  |
| (Aste) V 13824 | 2 | 3,00 | 2,00 | 1,00 | 1,00 |  |  |  |  |  |  |
| (Aste) V 10309 | 4 | . | . | . | . |  |  |  |  |  |  |
| An 2 | 1 | 4,00 | 1,00 | 1,00 | 1,00 |  |  |  |  |  |  |
| An 2 | 2 | 5,00 | 1,00 | 3,00 | 1,00 |  |  |  |  |  |  |
| An 2 | 3 | 3,00 | 1,00 | 2,00 | . |  |  |  |  |  |  |
| An 2 | 4 | 3,00 | 1,00 | 2,00 | . |  |  |  |  |  |  |
| (Caiapó x An 2) F_2_ | 1 | 4,70 | 1,00 | 1,00 | 1,40 |  |  |  |  |  |  |
| (Caiapó x An 2) F_2_ | 2 | 5,70 | 2,00 | 1,40 | 1,70 |  |  |  |  |  |  |
| (Caiapó x An 2) F_2_ | 3 | 2,70 | 2,00 | 2,00 | . |  |  |  |  |  |  |
| (Caiapó x An 2) F_2_ | 4 | 2,00 | 1,50 | 1,50 | . |  |  |  |  |  |  |
